# Nutritional Preconditioning of Apigenin Alleviates Myocardial Ischemia/Reperfusion Injury via the Mitochondrial Pathway Mediated by Notch1/Hes1

**DOI:** 10.1155/2019/7973098

**Published:** 2019-03-20

**Authors:** Huang Huang, Songqing Lai, Yong Luo, Qing Wan, Qicai Wu, Li Wan, Wanghong Qi, Jichun Liu

**Affiliations:** ^1^Department of Cardiac Surgery, The First Affiliated Hospital of Nanchang University, Nanchang, Jiangxi 330006, China; ^2^Jiangxi Provincial Key Laboratory of Women's Reproductive Health, Jiangxi Provincial Maternal and Child Health Hospital, Nanchang 330006, China; ^3^Department of Pharmacy, The First Affiliated Hospital of Nanchang University, Nanchang, Jiangxi 330006, China

## Abstract

Apigenin (Api), a natural flavone found in high amounts in several herbs, has shown potent cardioprotective effects in clinical studies, although the underlying mechanisms are not clear. We hypothesized that Api protects the myocardium from simulated ischemia/reperfusion (SI/R) injury via nutritional preconditioning (NPC). Rats fed with Api-containing food showed improvement in cardiac functions; lactate dehydrogenase (LDH) and creatine phosphokinase (CPK) activities; infarct size; apoptosis rates; malondialdehyde (MDA) levels; caspase-3, superoxide dismutase (SOD), glutathione peroxidase (GSH-Px), and catalase (CAT) activities; and ferric reducing antioxidant power (FRAP) compared to those fed standard chow following SI/R injury. In addition, Api pretreatment significantly improved the viability, decreased the LDH activity and intracellular reactive oxygen species (ROS) generation, alleviated the loss of mitochondrial membrane potential (MMP), prevented the opening of the mitochondrial permeability transition pore (mPTP), and decreased the caspase-3 activity, cytochrome c (Cyt C) release, and apoptosis induced by SI/R in primary cardiomyocytes. Mechanistically, Api upregulated Hes1 expression and was functionally neutralized by the Notch1 *γ*-secretase inhibitor GSI, as well as the mPTP opener atractyloside (Atr). Taken together, Api protected the myocardium against SI/R injury via the mitochondrial pathway mediated by the Notch1/Hes1 signaling pathway.

## 1. Introduction

Nutritional preconditioning (NPC), i.e., regular intake of natural nutrients as part of daily diet, has shown cardioprotective effects in various studies [1–4]. Several pharmacological and mechanical strategies have been developed to protect the myocardium against ischemia/reperfusion (I/R) injury [5], and they exert their ischemic pre- and postconditioning and cardioprotective effects by limiting calcium load and mPTP opening, reducing the generation of intracellular reactive oxygen species (ROS), and alleviating the loss of mitochondrial membrane potential (MMP) [6, 7].

Apigenin (Api) or C_15_H_10_O_5_, the aglycone of several naturally occurring glycosides, has several potent biological effects and has been used in traditional Chinese medicine (TCM) as a cardioprotective agent against I/R injury [8, 9]. Compared to ischemic pre- and postconditioning, NPC is more feasible as a long-term treatment. Previous studies have validated the protective effects of NPC with some TCMs such as ferulic acid and tanshinone IIA against myocardial I/R injury [2, 3]. We hypothesized that NPC by Api protected the myocardium against simulated ischemia/reperfusion- (SI/R-) induced damage and is aimed at elucidating the underlying mechanisms of these effects.

The Notch1 signaling pathway regulates multiple cellular processes like development, proliferation, differentiation, apoptosis, regeneration, and cell fate determination [10–12]. The Notch proteins exist as four homologs (Notch 1-4) in mammals and interact with a number of surface-bound or secreted ligands, such as Delta 1-4 and Jagged 1-2 [13, 14]. Following ligand binding, a disintegrin and metalloprotease (ADAM) and *γ*-secretases cleave and activate the Notch receptors [15], followed by nuclear translocation of the intracellular Notch domain and interaction with the recombination signal-binding protein for immunoglobulin J*κ* (RBP-J*κ*) region, which activates the downstream targets [16]. Hes1, a downstream target gene of the Notch1 signaling pathway, is known to protect cardiomyocytes by regulating the Notch1/Hes1-phosphatase and tensin homologue (PTEN)/Akt and phosphatidylinositol 3-kinase (PI3K)/Akt signaling pathways [17, 18]. The Notch1-Hes1 signaling pathway is also modulated by TCM formulations and compounds such as saponin astragaloside IV, the glycogen synthase kinase 3*β* (GSK3*β*) inhibitor indirubin-3′-monoxime, Xiaotan Sanjie decoction, salidroside, and Xinfeng capsule [19–23].

Based on the above, we hypothesized that NPC by Api could alleviate myocardial SI/R injury via the Notch1/Hes1 signaling pathway. To validate this hypothesis, we analyzed the protective effects of Api pretreatment on SI/R injury modeled on both isolated heart and cultured primary cardiomyocytes, and the underlying mechanisms.

## 2. Materials and Methods

### 2.1. Reagents

Apigenin (Api, purity > 98%) was purchased from the National Institute for the Control of Pharmaceutical and Biological Products (Beijing, China). GSI (*γ*-secretase inhibitor) was purchased from Selleckchem (S2215, USA) and atractyloside (Atr, mPTP opener) from Sigma (USA).

### 2.2. Animals

Adult and neonatal (2-3 days old) SD rats were purchased from the Animal Center of Nanchang University (Nanchang, China). The animal experiments were conducted as per the Guidelines for the Care and Use of Laboratory Animals (National Institutes of Health Publication No. 85-23, revised 1996) and approved by the Ethics Committee of Nanchang University.

The rats were housed two per cage in a controlled environment at 22°C, 50% humidity, and 12-hour light/dark cycle.

### 2.3. Experiments In Vivo

#### 2.3.1. Experimental Groups In Vivo

**Figure 1:**
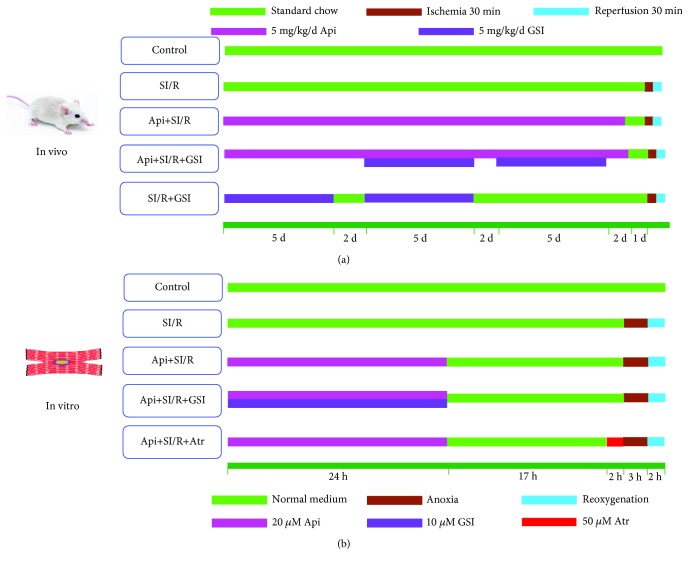
The schematic diagram for nutritional preconditioning using Api. (a) Rats were fed with 5 mg/kg body weight/day Api-containing chow daily for 3 weeks and then with standard chow 24 h before establishing the SI/R model. According to the pharmacokinetics theory, after 4-6 half-lives (approximately 4-6 hours), Api is cleared away completely in rats. (b) Cardiomyocytes were pretreated with 20 *μ*M Api for 24 h, and then normally cultured for 19 h prior to SI/R injury.

The adult SD rats were randomized into five groups and fed as follows (Figure 1(a)): (1) control group; (2) SI/R group: rats were fed standard chow for 3 weeks; (3) Api + SI/R group: 5 mg/kg body weight/day Api-containing chow for 3 weeks; (4) Api + SI/R + GSI group: 5 mg/kg body weight/day Api-containing chow for 3 weeks and intraperitoneally injected 5 mg/kg body weight/day GSI from the 2nd week (5 days on and 2 days off) as previously described [24]; and (5) SI/R + GSI group: intraperitoneally injected 5 mg/kg body weight/day GSI for 14 days (5 days on and 2 days off). The rats in the control, SI/R, and Api + SI/R groups were intraperitoneally injected with 5 mg/kg phosphate-buffered saline (PBS) daily. Twenty-four hours before SI/R injury, the diet was switched to standard chow in all animals.

#### 2.3.2. SI/R Model

The SI/R model was established as previously described [4]. Rats were anaesthetized by intraperitoneal injection of 30 mg/kg sodium pentobarbital, and after midline sternotomy, the hearts were excised and collected in Krebs-Henseleit (K-H) solution at 4°C. The hearts were then placed in a modified Langendorff apparatus and perfused with K-H solution (pH 7.4, NaCl 119 mM, NaHCO_3_ 25 mM, KCl 4.7 mM, KH_2_PO_4_ 1.2 mM, MgSO_4_·7H_2_O 1.2 mM, CaCl_2_ 2.5 mM, and glucose 11 mM) for 30 min at 37°C under constant pressure of 80 cmH_2_O and 95% O_2_/5% CO_2_. A water-filled latex balloon was inserted into the left ventricle by left arteriotomy, and the volume was adjusted to achieve a stable left ventricular end-diastolic pressure of 5 to 6 mmHg during initial equilibration. To induce ischemia, perfusion was continued with a modified glucose-free K-H solution for 30 min at 37°C, saturated with 95% N_2_/5% CO_2_ (pH 6.8). This was followed by a 30 min reperfusion with regular K-H solution. For the control group, the hearts were placed in the K-H solution throughout. We cut 3-5 mm (0.2-0.3 g) tissues from the apex of the heart for other particular measurements before hearts were cut and stained for infarct size measurement. The remaining tissues from the heart were cut into 0.8 mm thick transverse slices for 3-5 slices each heart to measure the myocardial infarct size.

#### 2.3.3. Measurement of Cardiac Function

The left ventricular pressure (LVP, kPa) and the maximal positive and negative change in the LVP (±dp/dtmax, kPa/s) of the isolated hearts (*n* = 10) were measured continuously using the PowerLab system (AD Instruments, Australia).

The activity of lactate dehydrogenase (LDH) and creatine phosphokinase (CPK) (*n* = 10) in the perfusate 5 min after the 30 min reperfusion period was determined using a Beckman automatic biochemical analyzer.

#### 2.3.4. Measurement of Biochemical Indices

The ferric reducing antioxidant power (FRAP), antioxidant enzyme activities, and lipid peroxidation level (*n* = 5) in myocardial homogenate were measured using specific kits for FRAP, MDA, SOD, CAT, and GSH-Px (Nanjing Jiancheng Bioengineering Institute, Nanjing, China) according to the manufacturer's guidelines. The absorbance of the supernatants was measured using a microplate reader.

#### 2.3.5. Measurement of Myocardial Infarct Size

The myocardial infarct size (*n* = 10) was measured as previously described [2]. Briefly, the hearts were removed from the Langendorff apparatus, weighed, and frozen at -20°C. The frozen tissues were cut into 0.8 mm thick transverse slices for 3-5 slices and incubated with 1% triphenyl tetrazolium chloride (TTC) for 30 min at 37°C. The stained slices were then fixed with 10% formaldehyde for 4-6 h at 22°C. The damaged area was calculated by subtracting the cavity-containing area from the total ventricular area, and the infarct size was represented as the percentage of the damaged area.

#### 2.3.6. Detection of Caspase-3 Activity

The myocardial tissues (*n* = 10) were homogenized, and the cytosolic fraction was resuspended in the lysis buffer and kept on ice for 15 min, followed by centrifugation at 4°C and 16,000 *g* for 15 min. Approximately 2 × 10^7^ cardiomyocytes (*n* = 8) were resuspended in the lysis buffer and kept on ice for 15 min. The supernatant was mixed with the specific detection buffer and Ac-DEVD-рNA provided with the caspase-3 activity assay kit (Beyotime, China) and incubated for 2 h at 37°C. The absorbance of the supernatants was measured at 405 nm.

#### 2.3.7. TUNEL Staining

Myocardial apoptosis (*n* = 10) was analyzed by the terminal deoxynucleotidyl transferase mediated nick end labelling (TUNEL) method. The left ventricular tissues containing the damaged areas were fixed in formalin for 24 h, embedded in paraffin, and cut into 5 *μ*m thick sections. After clearing the paraffin with xylene and rehydrating with an alcohol gradient, the sections were stained using a TUNEL kit (Promega, USA) according to the manufacturer's instructions. The apoptosis index was calculated as the percentage of TUNEL-positive cardiomyocytes.

#### 2.3.8. Western Blotting Assay

The myocardial tissues/cardiomyocytes were homogenized in a generic protein lysis buffer (Beyotime, China), and equal amounts of total protein per sample were separated by 12% sodium dodecyl sulfate polyacrylamide gel electrophoresis (SDS-PAGE) and transferred to nitrocellulose membranes (Millipore). The latter were blocked with 10% skim milk in TBST (Tris-buffered saline, 0.2% Tween-20) at room temperature for 2 h and then incubated overnight with anti-*β*-actin (Affinity, USA, 1 : 500) and anti-Hes1 (Abcam, USA, 1 : 500) or anti-Cyt C (Santa Cruz, USA, 1 : 500) antibodies at 4°C. The blots were then incubated with horseradish peroxidase- (HRP-) labeled immunoglobulin G (IgG) secondary antibodies (Zsgb Bio, China, 1 : 2000) for 2 h at room temperature. The positive bands were detected using Pierce enhanced chemiluminescence (ECL) Western blot substrate (Therom, USA) and imaged by ImageQuant LAS 4000 (GE, USA). Quantity One software (Bio-Rad, USA) was used for densitometric scanning of the bands, and specific protein levels were calculated relative to that of *β*-actin.

### 2.4. Experiments In Vitro

#### 2.4.1. Cardiomyocyte Culture

Neonatal (2-3 days old) SD rats were sacrificed, and their cardiomyocytes were isolated as previously described [25]. Briefly, the ventricles were separated and digested with 0.1% trypsin and centrifuged at 60 *g* for 5 min. The pellet was resuspended in Dulbecco's modified Eagle medium (DMEM, with 15% fetal bovine serum and 100 U/ml of penicillin and streptomycin), and the cells were plated on 60 mm culture dishes. After incubating for 2 h to remove nonmyocytes, the supernatant was collected and the enriched myocytes were plated on 60 mm gelatin-coated culture dishes at the density of 1 × 10^6^ cells per dish. After 24 h of culture, the cardiomyocytes were washed and a fresh medium was added.

#### 2.4.2. In Vitro Simulated Ischemia/Reperfusion (SI/R) Modeling in Cardiomyocytes

To induce SI/R injury in the cardiomyocytes, they were normally cultured for 42 h and then with fresh ischemic medium (NaH_2_PO_4_ 0.9 mM, NaHCO_3_ 6 mM, CaCl_2_ 1.8 mM, MgSO_4_ 1.2 mM, sodium lactate 40 mM, HEPES 20 mM, NaCl 98.5 mM, and KCl 10 mM, pH 6.8) for 3 h at 37°C under 95% N_2_ and 5% CO_2_. The ischemic medium was then replaced with the reperfusion medium (NaCl 129.5 mM, KCl 5 mM, NaH_2_PO_4_ 0.9 mM, NaHCO_3_ 20 mM, CaCl_2_ 1.8 mM, MgSO_4_ 1.2 mM, glucose 5.5 mM, and HEPES 20 mM, pH 7.4), and the cells were incubated for 2 h at 37°C under 95% O_2_ and 5% CO_2_.

#### 2.4.3. Experimental Groups In Vitro

The primary cardiomyocytes were randomized into five groups (in Figure 1(b)) and treated as follows: (1) control group: normal medium under normoxic conditions with 5% CO_2_ for 48 h, (2) SI/R group: subjected to SI/R as described above, (3) Api + SI/R group: 24 h treatment with different concentrations of Api (10, 20, 40, or 80 *μ*M), followed by 19 h normal culture and then SI/R injury, (4) Api + SI/R + GSI group: 24 h treatment with 20 *μ*M Api and 10 *μ*M GSI, followed by 19 h normal culture and then SI/R injury, and (5) Api + SI/R + Atr group: 24 h treatment with 20 *μ*M Api, normal culture for 17 h, 2 h incubation with 50 *μ*M Atr, and then SI/R injury.

#### 2.4.4. MTT Assay and Detection of LDH Activity

Viability of cardiomyocytes was evaluated by the 3-(4,5-dimethylthiazol-2-yl)-5-(3-carboxymethoxyphenyl)-2-(4-sulfophenyl)-2H-tetrazolium (MTT) assay (*n* = 8). Briefly, 1 × 10^4^ cardiomyocytes were plated per well in 96-well plates and, after SI/R treatment, were incubated for 3 h with 20 *μ*l MTT (Promega, USA) in 100 *μ*l medium at 37°C according to the manufacturer's instructions. The absorbance of each well was measured at 490 nm using a microplate reader (Bio-Rad 680, USA).

Post SI/R treatment, the culture media were collected and LDH activity (*n* = 8) was measured using an assay kit (Jiancheng, China).

#### 2.4.5. Annexin V/PI Staining

Approximately 2 × 10^6^ cardiomyocytes were resuspended in 500 *μ*l binding buffer provided in the Annexin V/Propidium iodide (PI) apoptosis detection kit (BD Biosciences, USA). To each tube, 10 *μ*l Annexin V-FITC and 5 *μ*l PI were added, and the cells were incubated at room temperature for 15 min in the dark. The stained cells were analyzed by flow cytometry at excitation and emission wavelengths (ex/em) of 488 nm and 578 nm (Becton Dickinson, USA). The apoptosis rate was obtained by calculating the ratio of Annexin V-positive/PI-negative cells to the total number of viable cells (*n* = 8).

#### 2.4.6. Measurement of Mitochondrial Membrane Potential (MMP)

The MMP (*n* = 6) was assessed by the fluorescent dye 5,5′,6,6′-tetrachloro-1,1′,3,3′-tetraethyl-imidacarbocyanine (JC-1, Invitrogen, USA). Cardiomyocytes were incubated with 200 *μ*M JC-1 for 20 min at 37°C and washed twice with cold PBS. Fluorescence was assessed by flow cytometry (Becton Dickinson, USA) at an initial ex/em of 530 nm and 580 nm (red) and then at 485 nm and 530 nm (green), respectively. The MMP was calculated as the ratio of red-stained to green-stained cells.

#### 2.4.7. Measurement of Intracellular Reactive Oxygen Species (ROS) Generation

ROS levels (*n* = 6) were measured using 2,7-dichlorofluorescein diacetate (DCFH-DA), which is converted to DCFH2 by intracellular esterases, and then oxidized by the ROS into the highly fluorescent DCF. The cardiomyocytes were resuspended in DMEM and incubated with 10 *μ*M DCFH-DA (Invitrogen, USA) for 20 min at 37°C according to the manufacturer's instructions. Fluorescence was measured by flow cytometry (Becton Dickinson, USA) at ex/em of 485 and 528 nm.

#### 2.4.8. Evaluation of Mitochondrial Permeability Transition Pore (mPTP) Opening

Mitochondria of the cardiomyocytes were isolated using the mitochondrial/cytosolic fractionation kit (QIAGEN, Germany), and mPTP opening was measured by the Ca^2+^-induced mitochondria swelling assay (*n* = 6). Briefly, 40 *μ*l of 200 *μ*M CaCl_2_ was added to cause mitochondrial swelling, and mPTP opening was measured in terms of the changes in absorbance at 520 nm per min (ΔODmin^−1^).

### 2.5. Statistical Analysis

SPSS 18.0 was used for statistical analysis. All values were presented as mean ± S.E.M. One-way ANOVA was used to compare the biochemical indices of different groups. A *p* value less than 0.05 was considered statistically significant. At least five independent experiments were performed for each assay.

## 3. Results

### 3.1. Api Pretreatment Improved Cardiac Function and Protected Myocardium against SI/R Injury

**Figure 2:**
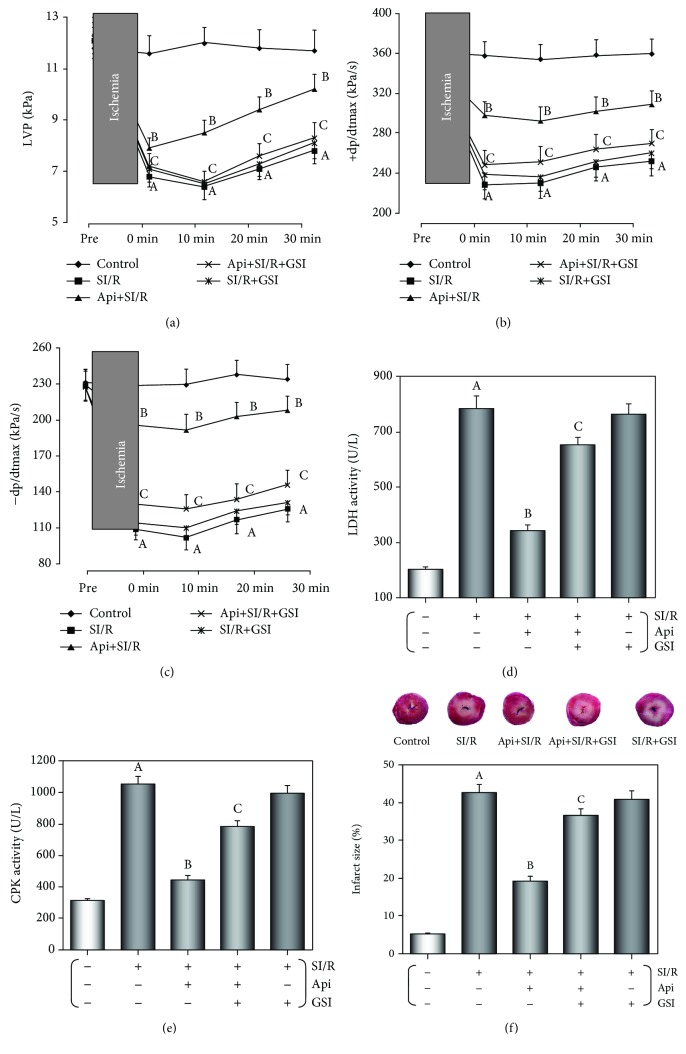
GSI abolished the protective effects of Api-pretreated on cardiac function, CPK and LDH activities, and infarct size against SI/R injury. (a–c) Cardiac function of isolated hearts in terms of LVP and ±dp/dtmax. (d, e) CPK and LDH activities in coronary flow effluent. (f) The infarct size was measured as the percentage of the damaged area. The values are presented as mean ± S.E.M. of 10 individual experiments. (a) *p* < 0.01 vs. control group; (b) *p* < 0.01 vs. SI/R group; (c) *p* < 0.01 vs. Api + SI/R group.

We evaluated the cardiac functions of the isolated hearts by recording their LVP and ±dp/dtmax (Figures 2(a)–2(c)). While the preischemic baseline parameters were similar across the different groups, 15 min of ischemia resulted in a significant drop in the LVP and ±dp/dtmax compared to the control group (*p* < 0.01) during reperfusion. However, both parameters were significantly higher in the Api + SI/R compared to the SI/R group (*p* < 0.01) and lower in the Api + SI/R + GSI compared to the Api + SI/R group (*p* < 0.01). In addition, the coronary flow effluent was collected after SI/R injury to evaluate the activities of CPK and LDH. As shown in Figures 2(d)–2(e), the activity of both enzymes was significantly increased after SI/R injury (*p* < 0.01) but were restored to near-normal levels by Api pretreatment (*p* < 0.01). However, CPK and LDH activities were significantly higher in the Api + SI/R + GSI compared to the Api + SI/R group (*p* < 0.01). Furthermore, the size of SI/R-induced infarction was significantly decreased in the Api + SI/R group (*p* < 0.01 vs SI/R group) and increased in the Api + SI/R + GSI group (*p* < 0.01 vs Api + SI/R group) (Figure 2(f)). Taken together, Api pretreatment protected the myocardium against I/R injury by improving the cardiac function, decreasing the LDH and CPK activities, and reducing the infarct size, and these protective effects were abrogated by GSI.

### 3.2. Api Pretreatment Inhibited Myocardial Apoptosis Resulting from SI/R Injury

**Figure 3:**
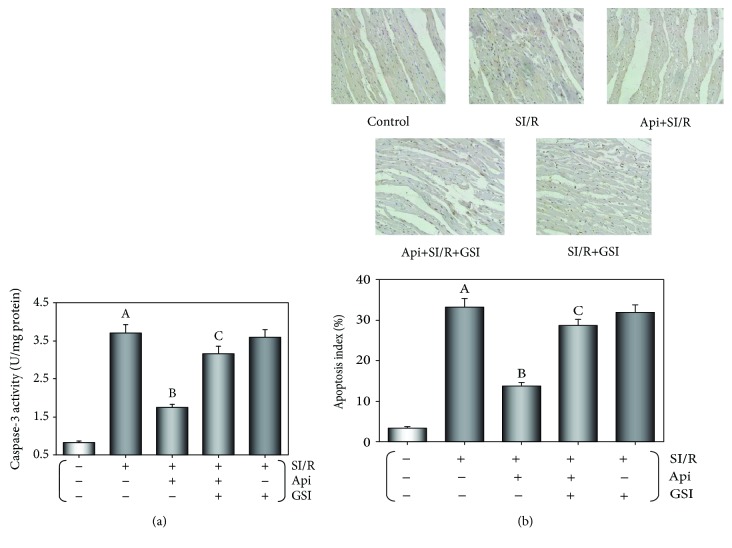
GSI abolished the protective effects of Api pretreatment on SI/R-induced myocardial apoptosis. (a) Bar graphs indicating caspase-3 activity in different groups. (b) The apoptosis index or percentage of TUNEL-positive cardiomyocytes. Values are presented as mean ± S.E.M. of 10 individual experiments. (a) *p* < 0.01 vs. control group; (b) *p* < 0.01 vs. SI/R group; (c) *p* < 0.01 vs. Api + SI/R group.

As shown in Figure 3(a), SI/R resulted in a significant increase in caspase-3 activity (*p* < 0.01), which was decreased following Api pretreatment and restored by including GSI during the NPC. Consistent with this, *in vitro* SI/R modeling significantly increased the apoptosis index or the percentage of TUNEL-positive cardiomyocytes (*p* < 0.01), which was reduced in the Api-pretreated cells (*p* < 0.01), and significantly increased in the Api + SI/R + GSI group (*p* < 0.01) (Figure 3(b)). Taken together, Api pretreatment protected against SI/R injury in the isolated hearts by decreasing apoptosis, which was reversed by GSI.

### 3.3. Api Pretreatment Alleviated SI/R Induced Oxidative Stress in the Myocardium

Oxidative stress damage in the myocardium following SI/R injury was measured in terms of lipid peroxidation levels, antioxidant enzyme activity, and FRAP (Table 1). Api pretreatment reversed the significant increase in MDA levels and the reduction in CAT, SOD, and GSH-Px activities and FRAP induced by SI/R (*p* < 0.01 for I/R vs control and Api + SI/R vs SI/R). However, unlike the other protective effects induced by Api pretreatment, oxidative stress damage was further decreased by GSI and Api cotreatment, indicating that the antioxidant effect of Api-pretreated was independent of the Notch1/Hes1 signaling pathway.

### 3.4. Api Pretreatment Upregulated Hes1 Expression in the Myocardium Undergoing SI/R Injury

**Figure 4:**
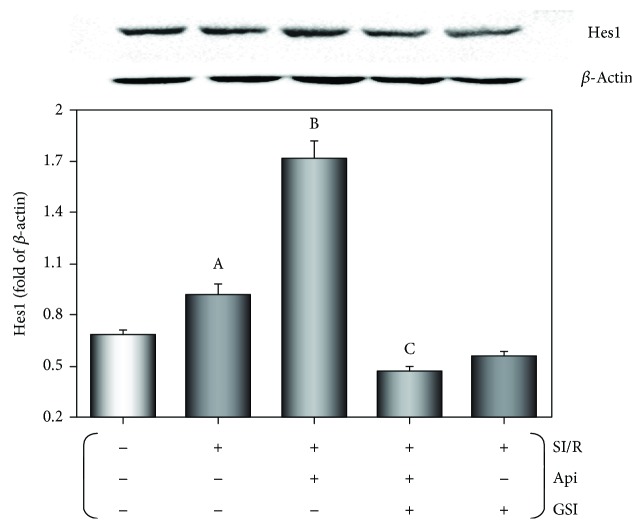
Effect of Api pretreatment on SI/R-induced Hes1 expression in the myocardium. Western blots showing Hes1 expression in the myocardium. Values are presented as mean ± S.E.M. of 10 individual experiments. (a) *p* < 0.01 vs. control group; (b) *p* < 0.01 vs. SI/R group; (c) *p* < 0.01 vs. Api + SI/R group.

As shown in Figure 4, the myocardial Hes1 expression was induced after SI/R injury (*p* < 0.01) and further upregulated in the Api-pretreated tissues (*p* < 0.01). GSI treatment significantly downregulated Hes1 levels in the Api + SI/R + GSI group relative to the Api + SI/R group (*p* < 0.01). Therefore, Api pretreatment might protect the myocardium from SI/R injury by upregulating Hes1.

### 3.5. Api Pretreatment Protected Cardiomyocytes Subjected to SI/R Injury

**Figure 5:**
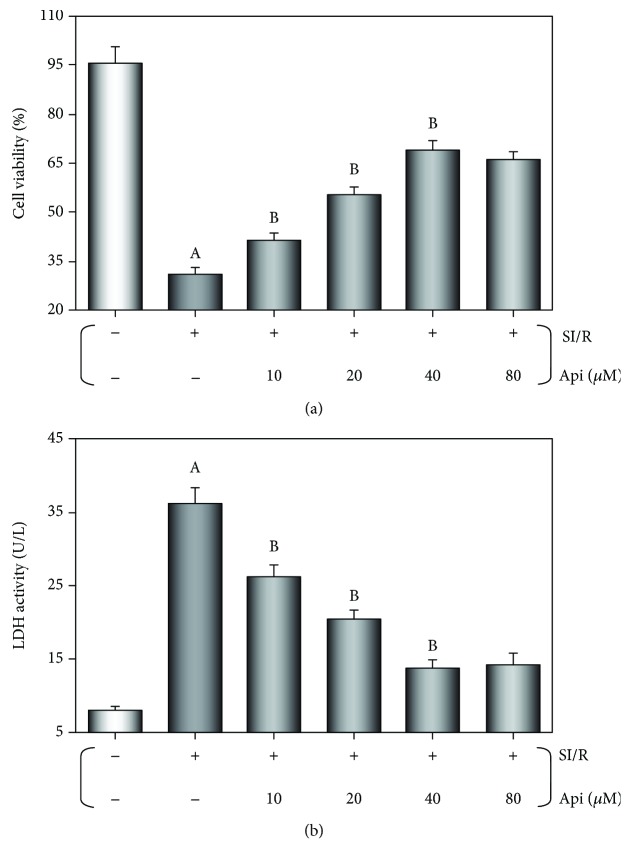
Effects of Api pretreatment on cardiomyocyte viability and LDH activity after SI/R injury. The primary cardiomyocytes were cultured with different concentrations of pretreated Api (10, 20, 40, and 80 *μ*M) for 24 h, then normally cultured for 19 h prior to SI/R injury. Api pretreatment increased cell viability (a) and reduced LDH activity (b) in a concentration-dependent manner. Values are presented as mean ± S.E.M. of 8 individual experiments. (a) *p* < 0.01 vs. control group; (b) *p* < 0.01 vs. SI/R group.

MTT assay was used to measure cardiomyocyte viability after SI/R injury. The significant reduction in cell viability following SI/R injury (*p* < 0.01) was alleviated by Api pretreatment in a dose-dependent manner (*p* < 0.01 vs. SI/R group) (Figure 5(a)). In addition, SI/R injury significantly increased LDH activity (*p* < 0.01) in the cardiomyocytes, which was restored to normal levels in the Api-pretreated cells (*p* < 0.01) in a concentration-dependent manner (Figure 5(b)). The cell viability was the highest, and the LDH activity was lowest with 40 *μ*M Api. According to the pharmacokinetics theory, the optimum concentration was calculated to be 20 *μ*M, which was used in the subsequent *in vitro* experiments.

**Figure 6:**
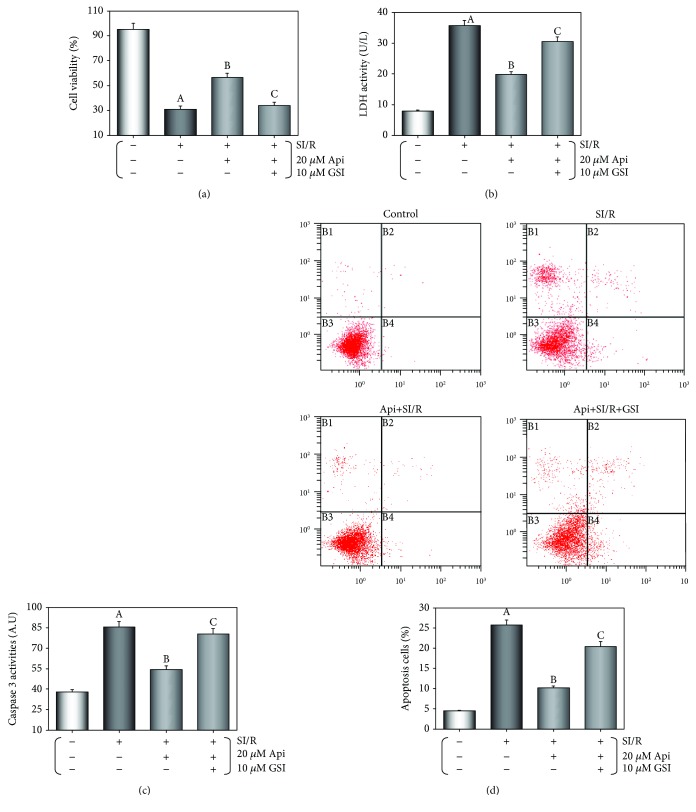
GSI abolished the protective effects of Api pretreatment on cardiomyocytes. (a) Cell viability was evaluated using the MTT assay. Column bar graphs showing LDH activity (b) and caspase-3 activity (c). (d) Representative flow cytometric dot plots (*x*-axis and *y*-axis representing Annexin V and PI staining, respectively) and the quantification of apoptotic cell population. Values are presented as mean ± S.E.M. of 8 individual experiments. (a) *p* < 0.01 vs. control group; (b) *p* < 0.01 vs. SI/R group; (c) *p* < 0.01 vs. Api + SI/R group.

As shown in Figures 6(a)–6(d), the cardiomyocytes subjected to SI/R injury had significantly reduced viability, increased LDH and caspase-3 activities, and apoptosis (*p* < 0.01 vs. control group), all of which were significantly reversed by 20 *μ*M in Api pretreatment (*p* < 0.01). The protective effects of Api pretreatment were attenuated by GSI cotreatment (*p* < 0.01).

### 3.6. Api Pretreatment Upregulated Hes1 Expression in Cardiomyocytes Subjected to SI/R Injury

**Figure 7:**
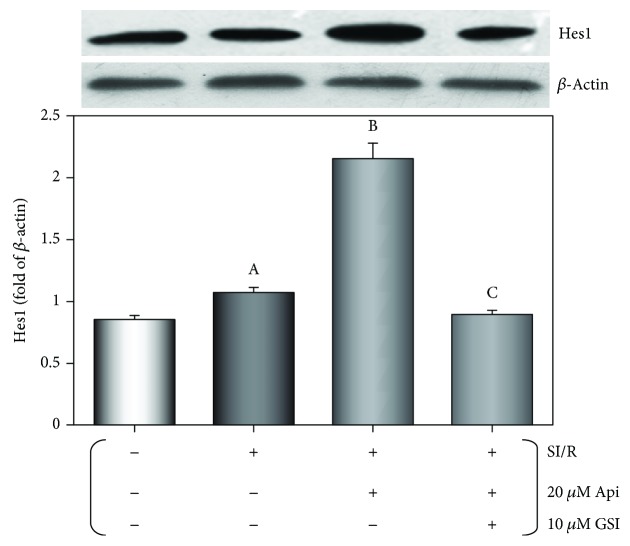
Effect of Api pretreatment on Hes1 expression in cardiomyocytes after SI/R injury. Western blots showing Hes1 protein levels in cardiomyocytes. Values are presented as mean ± S.E.M. of 6 individual experiments. (a) *p* < 0.01 vs. control group; (b) *p* < 0.01 vs. SI/R group; (c) *p* < 0.01 vs. Api + SI/R group.

As shown in Figure 7, SI/R injury induced Hes1 expression in the cardiomyocytes (*p* < 0.01), which was further upregulated in the Api + SI/R cardiomyocytes (*p* < 0.01) and significantly downregulated by GSI cotreatment, indicating the involvement of Hes1 in Api-mediated cardioprotection.

### 3.7. Api Pretreatment Maintained Mitochondrial Function in Cardiomyocytes Subjected to SI/R Injury

**Figure 8:**
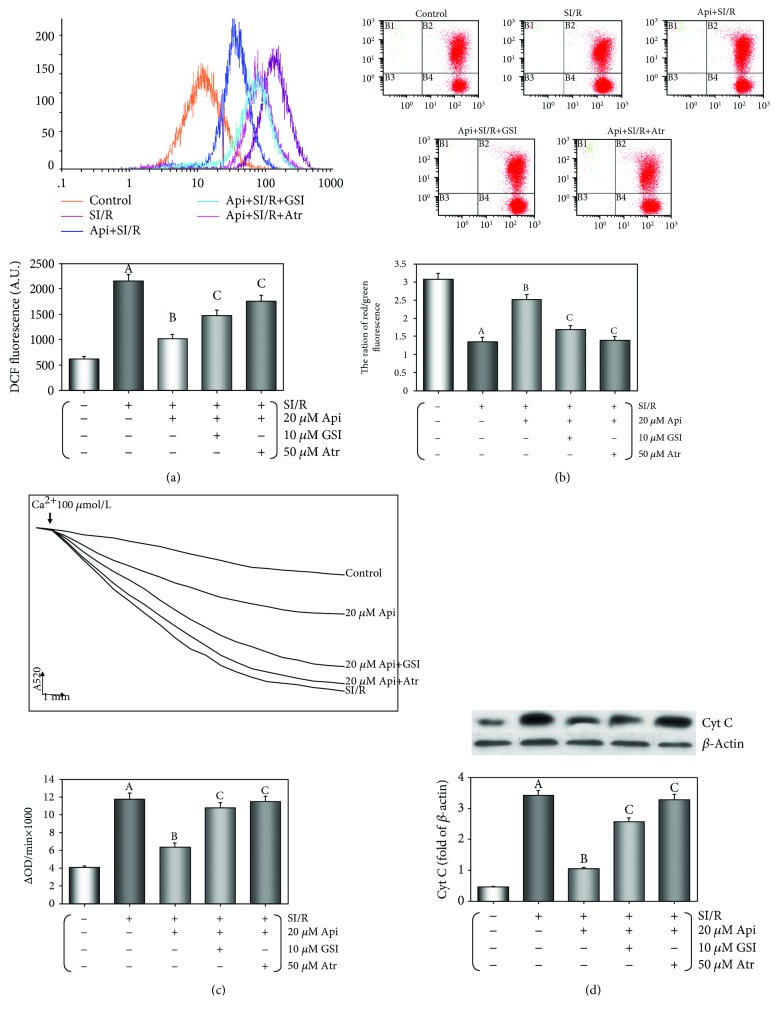
GSI or Atr abolished the protective effects of Api pretreatment on the maintenance of mitochondrial function after SI/R injury. (a) Flow cytometric histograms and column bar graph showing DCF fluorescence intensities. (b) The MMP levels evaluated by the ratio of fluorescence in the upper right quadrant and lower right quadrant. (c) The extent of mPTP opening evaluated as absorbance at 520 nm/min (ΔODmin^−1^) changes (ΔOD = A520_0min_ − A520_30min_). (d) Western blots showing cytosolic cytochrome c levels in cardiomyocytes. Values are presented as mean ± S.E.M. of 6 individual experiments. (a) *p* < 0.01 vs. control group; (b) *p* < 0.01 vs. SI/R group; (c) *p* < 0.01 vs. Api + SI/R group.

The mitochondrial function of cardiomyocytes was evaluated in terms of intracellular ROS generation, the loss of MMP, mPTP opening, and the release of cyt c into the cytosol. As shown in Figure 8(a), ROS generation was significantly increased following SI/R injury (*p* < 0.01 vs. control group) and reduced to normal levels in the Api-pretreated cells (*p* < 0.01). Cotreatment with GSI or Atr reversed the effect of Api pretreatment (*p* < 0.01), indicating that the Notch1/Hes1 pathway and mPTP opening were involved in Api-mediated ROS suppression.

Loss of MMP is an early sign of apoptosis and was evaluated by staining with the fluorescent cationic dye JC-1. The loss of MMP was calculated as the ratio of the fluorescence intensities of the upper right quadrant and lower right quadrant (Figure 8(b)). SI/R injury led to a significant reduction in this ratio and therefore a loss of MMP (*p* < 0.01 vs control group). Api pretreatment significantly attenuated MMP loss (*p* < 0.01 vs SI/R group), which reappeared in the GSI or Atr-administered cells (*p* < 0.01 vs Api + SI/R group) (Figure 8(b)), indicating that both GSI and Atr abolished the restorative effect of Api pretreatment on MMP.

As shown in Figure 8(c), ΔOD520, which is a measure of mPTP opening, was significantly increased after SI/R injury (*p* < 0.01 vs control group) and attenuated by Api pretreatment (*p* < 0.01 vs SI/R group). Both GSI and Atr significantly increased ΔOD520 (both *p* < 0.01 vs Api + SI/R group), indicating abrogation of the mPTP closing effect of Api pretreatment. As shown in Figure 8(d), SI/R injury significantly increased cyt c levels in the cytosol (*p* < 0.01), which was downregulated by Api pretreatment (*p* < 0.01 vs SI/R group) and reactivated by GSI and Atr (both *p* < 0.01 vs Api + SI/R group). Taken together, Api protected cardiomyocytes against SI/R injury by restoring mitochondrial functions via the Notch1/Hes1 pathway.

## 4. Discussion

Apigenin (Api), a flavone found in many plants, has been used as a drug in traditional Chinese medicine (TCM) to treat inflammation, cardiovascular disease, and cancer. It exhibits anti-inflammatory, antioxidative, and antiapoptotic effects and inhibits tumor cell invasion [26–29]. In our data, Api pretreatment improved the LVP and ±dp/dtmax; decreased CPK, LDH, and caspase-3 activities; and reduced the infarct size and apoptosis index of the myocardium, all of which protected against SI/R injury in the *in vivo* model. Api-pretreated cardiomyocytes showed a significant increase in viability and a decrease in LDH and caspase-3 activity and in apoptosis induced by SI/R injury. Abdukeyum et al. had proposed the concept of “nutritional preconditioning” (NPC), wherein natural nutrients obtained from daily diet can exhibit cardioprotective effects when used in small doses [1]. Taken together, NPC with Api induced cardioprotective effects. In addition, long-term NPC is more convenient than is pharmacological and anoxia preconditioning.

Several signaling pathways are involved in Api-induced cardioprotection, including the mitogen-activated protein kinases (MAPK) and sodium/glucose cotransporter 1 (SphK1)/sphingosine-1-phosphate (S1P) pathways [30, 31]. However, the mechanism underlying the long-term Api's NPC against SI/R injury is still unclear. The Notch1 signaling pathway is involved in a wide range of physiological processes, including cell survival, mitosis, transcriptional regulation, and metastasis [32–34]. In addition, the Notch1-Hes1 signaling pathway has been shown to protect cardiomyocytes from A/R injury [35–37]. We found that Hes1 was upregulated following both *in vivo* and *in vitro* SI/R injury, and the cardioprotective effects of Api were inhibited by the Notch1 *γ*-secretase inhibitor GSI, indicating the involvement of the Notch1/Hes1 signaling pathway in Api-mediated cardioprotection.

The Notch signaling pathway mediated the protective effects of curcumin on HUVECs against H_2_O_2_ by improving their adhesive and migratory abilities, increasing cell viability, and decreasing apoptosis and ROS generation [38]. Conversely, high ROS levels activated Notch signaling in SK-N-MC cells resulting in apoptosis, while EUK134 (a synthetic salen-manganese antioxidant complex) modulated the same pathway in these cells to prevent H_2_O_2_/menadione-induced death [39]. During myocardial ischemic postconditioning (IPost), Notch1 signaling is activated by hepatocyte growth factor (HGF) and ectopic expression of the Notch1 intracellular domain (N1ICD), which increases myocardial cell viability, reduces the loss of MMP, and prevents H9C2 cardiomyocyte apoptosis [40]. Furthermore, activated Notch1 signaling stabilized MMP and reduced ROS generation induced by I/R injury in H9C2 cells [41]. Based on these findings, we hypothesized that the Notch1/Hes1 signaling pathway in mitochondria was involved in the cardioprotective effects of Api's NPC against SI/R injury.

Excessive ROS generation and the resulting mitochondrial damage is an important mechanism underlying A/R [42]. ROS and Ca^2+^ are the by-products of normal mitochondrial metabolism, and their accumulation during disrupted mitochondrial function is potentially damaging. The activation of mPTP and the inner membrane anion channel mediate the adaptive and maladaptive responses to redox stress, leading to ROS release caused by intra- and intermitochondrial redox changes [43, 44]. Mitochondrial Ca^2+^ and ROS overload results in mPTP opening, leading to sarcolemmal rupture and necrotic cell death through ATP-dependent hypercontracture [45]. We analyzed oxidative stress damage by detecting the levels of lipid peroxidation, antioxidant enzyme activities, and FRAP. Api's NPC restored the levels of MDA, CAT, SOD, GSH-Px, and FRAP that were reduced in the myocardium following SI/R injury. This is consistent with previous studies that showed that Api increased SOD and GSH-Px levels and reduced ROS generation in different cells [46–48]. Interestingly, GSI further decreased oxidative stress damage, indicating that the antioxidant effect of Api might be independent of the Notch1/Hes1 signaling pathway. One study showed that GSI decreased ROS generation in N2a cells [49]. Taken together, the cardioprotection of Api involves reduction in oxidative stress via multiple signaling pathways that are independent of the Notch1/Hes1 pathway.

The breakdown of MMP, an early sign of apoptosis, is mediated by mPTP opening in the inner mitochondrial membrane, resulting in matrix swelling, outer membrane rupture, and the release of apoptotic signaling molecules during A/R [50]. The opening of mPTP is physiologically important and maintains mitochondrial homeostasis. Following cyt c release, caspase-9 is activated that leads to caspase-3 activation [51]. Caspase-3 is the key effector of mitochondria-mediated apoptosis [52]. In accordance with previous studies [47, 53–55], Api reduced caspase-3 activity and myocardial apoptosis, inhibited MMP loss and mPTP opening, and prevented cyt c release induced by SI/R. All these effects were abolished by GSI, indicating that protective effects of Api on cardiomyocytes against SI/R injury were mediated by Notch1/Hes1.

## 5. Conclusions

Api protected the myocardium against SI/R injury and reduced the infarct size by upregulating Hes1, improving cardiac function, increasing cardiomyocyte viability, inhibiting apoptosis and oxidative stress damage, and maintaining mitochondrial function. The cardioprotective effects of Api were abolished by both GSI (a Notch1 inhibitor) and Atr (a mPTP opener), indicating that Api exerted its effects via the Notch1-Hes1 signaling pathway. Regular intake of Api-rich foods could therefore result in nutritional preconditioning and prevent acute myocardial injury.

## Figures and Tables

**Table 1:** Api preserved reduced the levels of lipid peroxidation, the activities of antioxidant enzymes, and the ferric reducing antioxidant power in the myocardium against SI/R injury.

Groups	MDA content (nmol/g tissue)	CAT activity (U/g tissue)	SOD activity (U/g tissue)	GPx activity (U/g tissue)	FRAP (mmol Fe^2+^/L)
Control	31.6 ± 2.3	15.5 ± 1.2	85.1 ± 4.5	23.1 ± 1.5	5.29 ± 0.26
SI/R	135.2 ± 12.8^a^	3.8 ± 0.4^a^	20.6 ± 2.3^a^	6.1 ± 0.5^a^	1.32 ± 0.04^a^
Api + SI/R	51.5 ± 4.8^b^	13.1 ± 1.1^b^	63.1 ± 4.2^b^	18.2 ± 1.5^b^	4.61 ± 0.28^b^
Api + SI/R + GSI	66.3 ± 6.2	11.9 ± 1.2	60.4 ± 3.9	17.1 ± 1.8	3.81 ± 0.23
SI/R + GSI	82.1 ± 7.2	9.8 ± 0.8	57.3 ± 4.1	16.0 ± 1.5	1.53 ± 0.09

Values are presented as mean ± S.E.M. of five individual experiments. (a) *p* < 0.01 vs. control group; (b) *p* < 0.01 vs. SI/R group.

## Data Availability

The data used to support the findings of this study are available from the corresponding author upon request.

## Abbreviations

ADAM: A disintegrin and metalloprotease
Api: Apigenin
Atr: Atractyloside
A/R: Anoxia/reoxygenation
CAT: Catalase
CPK: Creatine phosphate kinase
Cyt C: Cytochrome C
DCFH-DA: 2,7-Dichlorofluorescein diacetate
ECL: Enhanced chemiluminescence
ex/em: Excitation and emission wavelengths
FITC: Fluorescein isothiocyanate
FRAP: Ferric reducing antioxidant power
GSH-Px: Glutathione peroxidase
GSI: *γ*-Secretase inhibitor
GSK3*β*: Glycogen synthase kinase 3*β*
HGF: Hepatocyte growth factor
HRP: Horseradish peroxidase
IgG: Immunoglobulin G
IPost: Ischemic postconditioning
I/R: Ischemia/reperfusion
JC-1: 5,5′,6,6′-Tetrachloro-1,1′,3,3′-tetraethyl-imidacarbocyanine
K-H: Krebs–Henseleit
LDH: Lactate dehydrogenase
LVP: Left ventricular pressure
MAPKs: Mitogen-activated protein kinases
MDA: Malondialdehyde
MMP: Mitochondrial membrane potential
mPTP: Mitochondrial permeability transition pore
MTT: 3-(4,5-Dimethylthiazol-2-yl)-5-(3-carboxymethoxyphenyl)-2-(4-sulfophenyl)-2H-tetrazolium
N1ICD: Notch1 intracellular domain
NPC: Nutrition preconditioning
PBS: Phosphate-buffered saline
pNA: p-Nitroaniline
PI: Propidium iodide
PI3K: Phosphatidylinositol 3-kinase
PTEN: Phosphatase and tensin homologue
RBP-J*κ*: Recombination signal binding protein for immunoglobulin J*κ*
ROS: Reactive oxygen species
SI/R: Simulated ischemia/reperfusion
SD: Sprague-Dawley
SDS-PAGE: Sodium dodecyl sulfate polyacrylamide gel electrophoresis
SGLT1: Sodium/glucose cotransporter 1
SOD: Superoxide dismutase
S1P: Sphingosine 1-phosphate
TBST: Tris-buffered saline, 0.2% Tween-20
TCM: Traditional Chinese medicine
TTC: Triphenyl tetrazolium chloride
TUNEL: Terminal deoxynucleotidyl transferase mediated nick end labelling
±dp/dtmax: Maximal positive and negative change in the LVP.

## Supplementary Materials

Supplementary Materials Figure S1: effects of Api pretreatment with or without coadministration of GSI or Atr on primary cardiomyocyte viability and LDH activity after SI/R. Cell viability was similar in the 20 *μ*M Api, 10 *μ*M GSI, 20 *μ*M Api + 10 *μ*M GSI, and 20 *μ*M Api + 50 *μ*M Atr groups compared to the control group and between the 10 *μ*M GSI + SI/R and SI/R groups. Values are mean ± S.E.M. of 6 individual experiments. (a) *p* < 0.01 vs. control group; (b) *p* < 0.01 vs. 50 *μ*M Atr group; (c) *p* < 0.01 vs. SI/R group. Figure S2: effects of Api pretreatment with or without coadministration of GSI or Atr on LDH activity in the primary cardiomyocytes after SI/R. LDH activity was similar in the 20 *μ*M Api, 10 *μ*M GSI, 20 *μ*M Api + 10 *μ*M GSI, and 20 *μ*M Api + 50 *μ*M Atr groups compared to the control group and between the 10 *μ*M GSI + SI/R and SI/R groups. Values are mean ± S.E.M. of 6 individual experiments. (a) *p* < 0.01 vs. control group; (b) *p* < 0.01 vs. 50 *μ*M Atr group; (c) *p* < 0.01 vs. SI/R group.
