# Supplementary material for: Nutritional Preconditioning of Apigenin Alleviates Myocardial Ischemia/Reperfusion Injury via the Mitochondrial Pathway Mediated by Notch1/Hes1
Source: Oxid Med Cell Longev. 2019 Mar 20;2019:7973098. doi: 10.1155/2019/7973098 (PMC6446095; doi:10.1155/2019/7973098)
Supplement: Supplementary Materials — Figure S1: effects of Api pretreatment with or without coadministration of GSI or Atr on primary cardiomyocyte viability and LDH activity after SI/R. Cell viability was similar in the 20 μM Api, 10 μM GSI, 20 μM Api + 10 μM GSI, and 20 μM Api + 50 μM Atr groups compared to the control group and between the 10 μM GSI + SI/R and SI/R groups. Values are mean ± S.E.M. of 6 individual experiments. (a) p < 0.01 vs. control group; (b) p < 0.01 vs. 50 μM Atr group; (c) p < 0.01 vs. SI/R group. Figure S2: effects of Api pretreatment with or without coadministration of GSI or Atr on LDH activity in the primary cardiomyocytes after SI/R. LDH activity was similar in the 20 μM Api, 10 μM GSI, 20 μM Api + 10 μM GSI, and 20 μM Api + 50 μM Atr groups compared to the control group and between the 10 μM GSI + SI/R and SI/R groups. Values are mean ± S.E.M. of 6 individual experiments. (a) p < 0.01 vs. control group; (b) p < 0.01 vs. 50 μM Atr group; (c) p < 0.01 vs. SI/R group. [file 7973098.f1.pdf]

Nutritional preconditioning of Apigenin alleviates myocardial ischemia/reperfusion injury via mitochondrial pathway mediated by Notch1 /Hes1

Supplementary Materials

Figure S1. Effects of Api pre-treatment with or without co-administration of GSI or Atr on primary cardiomyocyte viability and LDH activity after SI/R.

Cell viability was similar in the 20μM Api, 10μM GSI, 20μM Api+10μM GSI and 20μM Api+50μM Atr groups compared to control group, and between the 10μM GSI+SI/R and SI/R groups. Values are mean ± S.E.M. of 6 individual experiments. (a) $p < 0.01$ vs. control group; (b) $p < 0.01$ vs. 50μM Atr group; (c) $p < 0.01$ vs. SI/R group.

Figure S2. Effects of Api pre-treatment with or without co-administration of GSI or Atr on LDH activity in the primary cardiomyocytes after SI/R.

LDH activity was similar in the 20μM Api, 10μM GSI, 20μM Api+10μM GSI and 20μM Api+50μM Atr groups compared to the control group, and between the 10μM GSI+SI/R and SI/R groups. Values are mean ± S.E.M. of 6 individual experiments. (a) $p < 0.01$ vs. control group; (b) $p < 0.01$ vs. 50 μM Atr group; (c) $p < 0.01$ vs. SI/R group. (Supplementary Materials).

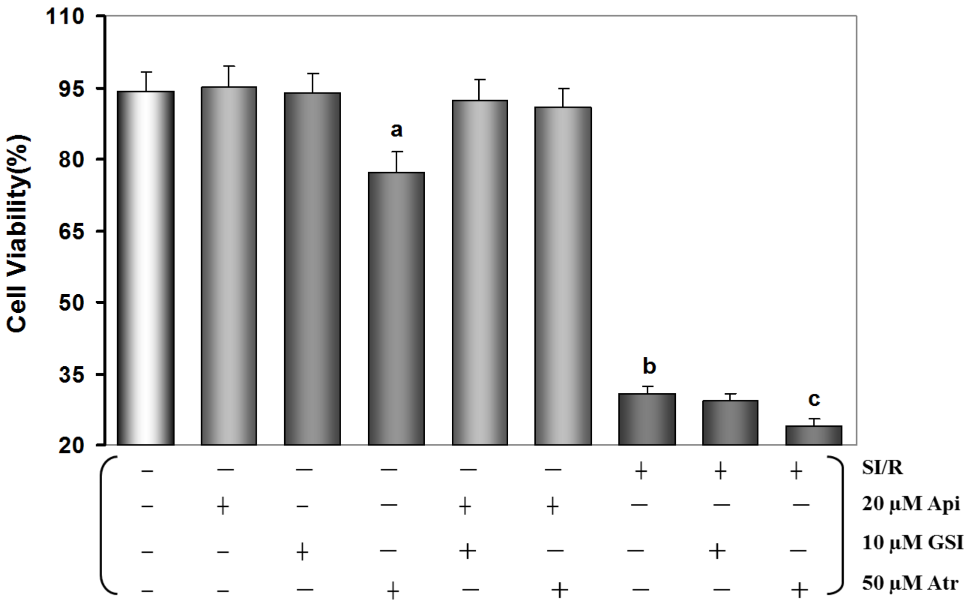

Figure S1

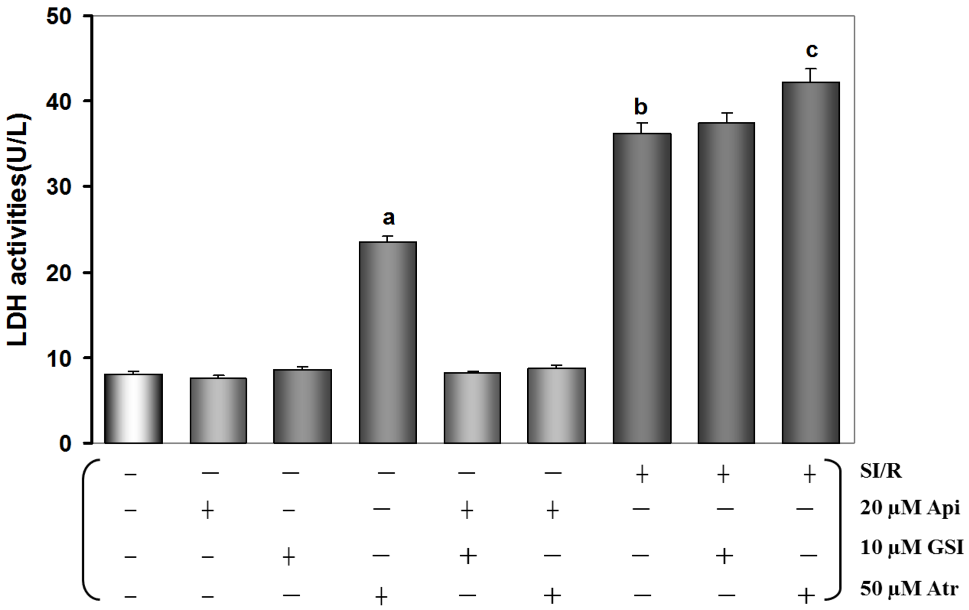

Figure S2
